# Knowledge, Attitude, and Practice of Diabetic Foot Ulcer Care in Asser Region: A Cross-Sectional Study

**DOI:** 10.7759/cureus.42807

**Published:** 2023-08-01

**Authors:** Ayoub A Alshaikh, Hassan M Alqarni, Hassan A Hassan Assiri, Mohammed A Shlwan, Mohammed A AlJebreel, Abdulrahman S Almuaddi, Mohammed A Asiri, Faisal Naser A Almuidh, Nawaf Y Al Qasim, Omar A Alshahrani, Ramy M Ghazy

**Affiliations:** 1 Family & Community Medicine, King Khalid University, Abha, SAU; 2 Al-Mahala Primary Health Care Center, Ministry of Health, Abha, SAU; 3 Medical School, King Khalid University, Abha, SAU; 4 Department of Public Health, Alexandria University, Alexandria, EGY

**Keywords:** knowledge attitude and practice, saudi arabia, diabetic foot ulcer management, public health education, diabetic foot education program, diabetes mellitus

## Abstract

Background

Foot problems continue to be the leading cause of hospital admissions among people with diabetes. The objective of this study was to explore and assess the knowledge and attitudes of individuals about diabetic foot ulcers in the Asser region, Saudi Arabia.

Methodology

An anonymous, online, cross-sectional survey was conducted. The questionnaire was distributed through commonly used social media platforms such as Instagram, Facebook, and Twitter.

Results

A total of 445 participants were included in this survey. Overall, 37.1% (165) were aged 18-25 years, 64.3% (286) were women, and 75.7% (337) had university-level education. A significant number of participants had relatives with diabetes (57.1%, 254), while a smaller percentage reported having diabetes themselves (7.3%, 33), and a substantial proportion were neither diabetic nor had a relative with diabetes (35.6%, (158). Nearly two-fifths of the participants (37.8%, 168) received information about diabetes and diabetic foot care from physicians, and 34.1% (152) of the participants accessed information online. There were significant differences between those who did not have diabetes mellitus (DM) and those who had DM or whose relatives were diabetic in responses to the following questions: “Do you think that diabetes may cause gangrene in the foot?” (50.9% (205) vs. 45.7% (32), p = 0.019), “Do you think that preventing diabetic foot ulcers is more important than treating diabetic foot ulcers?” (60.8% (228) vs. 46.9% (60), p = 0.002), and “Do you think it is important to constantly monitor diabetic foot wounds?” (63.1% (200) vs. 30.4% (17), p < 0.001). There was a statistically significant difference between groups in the practice of daily foot checks, washing feet, moisturizing feet, keeping feet away from hot and cold, and nail care (p < 0.001).

Conclusions

The participants in the study showed a lack of knowledge regarding diabetic foot care, indicating the potential for better outcomes through the implementation of enhanced health education programs.

## Introduction

Saudi Arabia has the second-highest prevalence of diabetes mellitus (DM) among Middle Eastern countries and ranks seventh globally [1]. A comprehensive epidemiological health survey was conducted throughout Saudi Arabia, targeting individuals between the ages of 30 and 70 years who lived in selected households. Of the 16,917 participants included in the survey, 4,004 were diagnosed with DM, representing approximately 23.7% of the population [2]. However, higher prevalence rates for DM have been reported among Saudi individuals, varying from 26.0% to 61.8% [3,4].

Foot problems continue to be the leading cause of hospital admissions among people with diabetes [5]. There is a 25% chance that a diabetic patient will develop an ulcer at some point in their lifetime, with more than 50% of these ulcers becoming infected and requiring hospitalization. This is associated with a significant chance of lower limb amputations [6,7]. These complications associated with diabetic foot conditions not only negatively impact patients’ health and quality of life but also contribute to higher mortality rates. This situation adds to the physical, psychological, and financial burdens faced by individuals with diabetes and their communities [8].

In the past 20 years, there has been a significant emphasis on studying the knowledge, attitudes, and practices (KAP) of individuals with DM concerning the care of their feet [9,10]. The primary objectives of these studies were to identify barriers to seeking medical care, improve self-care practices, and promote lifestyle changes among diabetics. The findings of these studies revealed a correlated and predictive relationship between KAP and diabetic foot. Although there may be some inconsistencies in the research findings, most studies support the notion that a higher level of knowledge and a positive attitude toward diabetic foot care lead to improved practices in the management of diabetic foot conditions [3,11]. In Riyadh, a study was conducted to assess the KAP of diabetic foot ulcers. The results showed that a significant majority of patients had good knowledge about diabetic foot and foot ulcers. Most patients washed their feet daily and checked their shoes before wearing them. However, less than half of the patients performed daily foot inspections. Only a small percentage of patients attended a diabetic foot care class or received education from physicians or nurses [12]. This study aims to explore and assess the KAP of individuals with or without DM regarding the diabetic foot in the Asser region. Ultimately, this study aims to contribute to the body of knowledge on diabetic foot ulcer care by shedding light on the specific knowledge gaps and attitudes that exist in the region. In addition, we aim to identify the main source of information of such knowledge.

## Materials and methods

Study setting and design

In May 2023, a cross-sectional study was conducted to evaluate the KAP of the population in the Asser region of Saudi Arabia regarding diabetic foot ulcers. To collect data, an anonymous electronic Google Forms questionnaire was created in Arabic and English. The questionnaire was distributed through commonly used social media platforms such as Instagram, Facebook, and Twitter.

Sample size, inclusion criteria, and sampling method

Using a 5% margin of error, 95% confidence level, and an assumed 50% of the population having good knowledge and attitude toward diabetic foot ulcer, a minimum sample size of 384 parents was calculated using Epi-info 7.2. We rounded it to 450 to compensate for non-response (10%) and incomplete and inconsistent data. The study population comprised individuals who met specific inclusion criteria, including willingness to participate, both genders, adults 18 years and older, having access to the Internet through a mobile phone or computer, having basic reading and writing skills, and residing in the Asser region.

The sample size was calculated using the following equation: (N) = ((Z2 × P × (1-P))/(E)^2)/expected response rate, where Z represents the standard normal distribution, which is 1.96 for a 95% confidence interval. P represents the proportion, which is 0.45 for vaccine acceptance in Libya. E represents the margin of error, which is 0.03. The expected response rate is 10%. Thus, the calculated sample size (N) was (N) = (1.96^2 × 0.50 × 0.45)/(0.03^2/0.1). Data collection for this study used a snowball sampling approach.

Data collection

The data collection questionnaire was meticulously crafted through a combination of a literature review and the incorporation of relevant questions from similar studies. Using this approach, we ensured that the questionnaire content reflected the most pertinent aspects related to our research. To establish the validity of the questionnaire, the researchers conducted both content and face validity testing. Content validity was ensured through an in-depth assessment of the questionnaire’s relevance, clarity, and comprehensiveness, with adjustments made based on expert feedback and insights from domain experts. Face validity testing involved piloting the questionnaire with a small sample of respondents to gauge their understanding of the questions and identify any potential ambiguities or confusion. Feedback from the pilot study was considered to refine the questionnaire and ensure its clarity and suitability for the study population. Furthermore, researchers evaluated the internal consistency of the developed tool using Cronbach’s alpha. The obtained Cronbach’s alpha coefficient was 0.86, indicating a high level of internal consistency among the items in the questionnaire. Each team member distributed the questionnaire to a minimum of 5-10 individuals to gauge the time needed for completion and identify potential issues. Based on the results of the pilot study, it was determined that the questionnaire could be completed within 5-10 minutes. Minor modifications were made to improve the clarity of certain sentences. Furthermore, measures were implemented to prevent duplicate submissions, guaranteeing that each participant could submit only one response. The questionnaire consisted of four distinct sections, each focusing on specific aspects related to diabetic foot care. The first section collected sociodemographic data, such as age, gender, and educational background. In the second section, participants were queried about diabetes-related factors, including their diabetic status, type of diabetes, and duration since diagnosis. The third section aimed to assess participants’ sources of information about diabetic foot care, encompassing avenues such as medical professionals, the internet, friends, and medical articles. Finally, the fourth section explored the KAP of the participants about possible complications associated with diabetes, such as foot ulcers, gangrene, and amputation. It also investigated whether participants sought specialized diabetic foot care and received explanations about foot care procedures while evaluating their perception of the importance of preventive measures and regular monitoring of foot wounds. It also evaluated daily foot checks, meticulous foot washing, application of moisturizers, examination of shoes before wearing, protection against extreme temperatures, and appropriate toenail care.

Ethical considerations

The study received approval from the Research Ethics Committee of King Khalid University (approval number: HAPO-06-B-001). Ethical consent was obtained and presented as an initial requirement in the questionnaire. Participants had the option to either provide their consent and complete the questionnaire or decline to participate. All personal information from participants was treated strictly confidential and kept anonymous throughout the study.

Statistical analysis

The statistical analysis was performed using SPSS version 27 (IBM Corp., Armonk, NY, USA). Categorical variables were presented as counts and percentages to summarize the data. Pearson’s chi-square test was utilized to compare the two independent categorical variables and determine any significant associations. Participants were categorized into the diabetic group as those who had either type 1 DM or type 2 DM or provided care to first-degree relatives complaining of DM. The other group was not diabetic and did not have first-degree relatives suffering from diabetes. In this case, the variables are the responses to the questions and the gender of the respondents. A p-value of 0.05 or less was considered statistically significant, indicating a meaningful relationship between the variables being compared.

## Results

Table 1 presents the distribution of the participants based on various demographic characteristics and variables related to DM. Regarding age, the majority of the participants were in the age groups of 18-25 years (37.1%, 165) and 36-50 years (31.9%, 142), while the lowest representation was observed in the age group of 51-65 years (8.3%, 37). In terms of gender, the sample consisted of predominantly women participants (64.3%, 286) compared to male participants (35.7%, 159). A small proportion of the participants had primary (0.4%, 2) or preparatory education (0.7%, 3), while the majority had secondary education (23.1%, 103) or university-level education (75.7%, 337). Regarding diabetes status, a significant number of participants had relatives with DM (57.1%, 254), while a smaller percentage reported having diabetes themselves (7.3%, 33), and a substantial proportion were neither diabetic nor had a relative with diabetes (35.6%, 158). According to the relatives’ diabetes category, the highest proportion was mothers or fathers (82.8%, 210), followed by siblings (11.4%, 29), sons or daughters (3.9%, 10), and housewives or husbands (1.9%, 5). Participants were mainly categorized as either type 1 (45.3%, 130) or type 2 (42.9%, 123), while a smaller proportion indicated uncertainty regarding the diabetes type (11.8%, 34).

**Table 1:** Demographic characteristics and diabetes profile of study participants (n = 445).

Variable	Demographic characteristics	Total, n (%)
Age (year)	18–25	165 (37.1%)
	26–35	101 (22.7%)
	36–50	142 (31.9%)
	51–65	37 (8.3%)
Sex	Female	286 (64.3%)
	Male	159 (35.7%)
Having diabetes mellitus	Primary	2 (0.4%)
	Preparatory	3 (0.7%)
	Secondary	103 (23.1%)
	University or higher education	337 (75.7%)
Having diabetes mellitus	Having diabetes mellitus	33 (7.3%)
	Having relatives’ diabetes mellitus	254 (55.8%)
	No diabetes	158 (35.7%)
Relatives’ diabetes mellitus (n = 254)	Sister or brother	29 (11.4%)
	Mother or father	210 (82.8%)
	Housewife or husband	5 (1.9%)
	Son or daughter	10 (3.9%)
Type of diabetes (n = 287)	Type 1	130 (45.3%)
	Type 2	123 (42.9%)
	Don’t know	34 (11.8%)
Duration of diabetes (n = 287)	Less than 6 months	31 (10.8%)
	6 months to 5 years	37 (12.9%)
	More than 5 years	156 (54.4%)
	Since birth	25 (8.7%)
	Don’t know	38 (13.2%)

**Figure 1:**
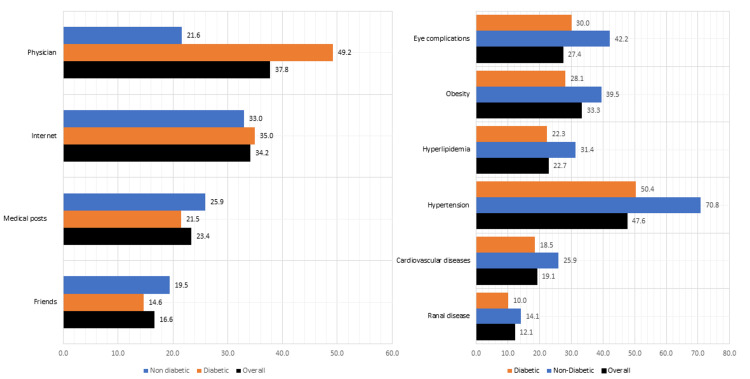
(a) Sources of Information about diabetic foot care among study participants (b) Respondents’ perspectives about the chronic diseases that are associated with diabetes mellitus.

Figure 1a depicts the different sources of information about diabetic foot care among the study participants. Nearly two-fifths of participants (37.7%, 168/445) received information on diabetic foot care from physicians, ((21.6%, 40/185) of non-diabetic vs. (49.2%, 128/260) of diabetic) and 34.1% (152/445%) of participants accessed information online ((32.9%, 61/185) of non-diabetic vs. (35.0%, 91/260) of diabetic). Medical posts and friends were also mentioned as sources of information with 23.4% (104/445) (25.9% (48/158) of non-diabetic vs. 21.5% (56/260) of diabetic) and 16.6% (74/445) (19.5% (36/185) of non-diabetic vs. 14.6% (38/260) of diabetic) of participants, respectively. Figure 1b presents the perception of the association between chronic diseases and DM. It indicates the percentage of respondents who believed that each listed chronic disease is associated with DM. The findings revealed that a significant proportion of respondents perceived eye complications (27.4%, 122/445), obesity (33.3%, 148/455), hyperlipidemia (22.7%, 101), hypertension (47.6%, 212/454), cardiovascular disease (19.1% 85/445), and renal disease (12.1%, 54/445) to be associated with DM.

**Figure 2:**
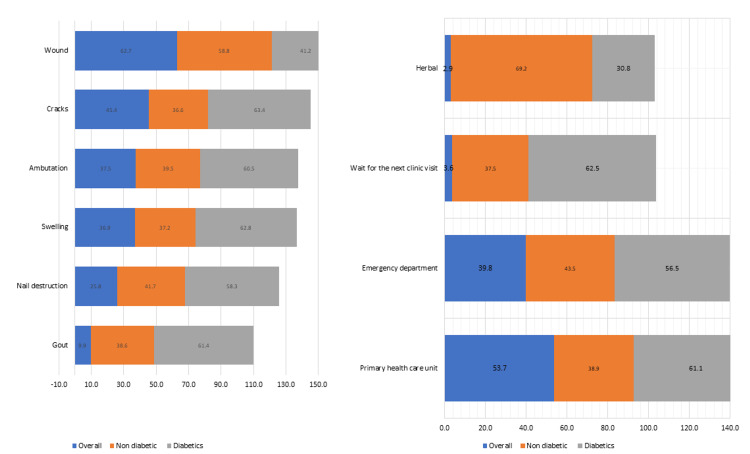
(a) Respondents’ answers about where to seek medical service related to diabetic foot. (b) Respondents’ perspectives about the complications that are associated with diabetic foot.

Figure 2a reveals that a substantial percentage of participants recognized the following complications: swelling at 36.9% (37.2% among non-diabetic vs. 62.80% diabetics), nail destruction at 25.8% (41.74% non-diabetics vs. 58.26 % diabetics), cracks at 45.4% (36.63% non-diabetics vs. 63.37% diabetics), and wounds at 62.7% (58.78% non-diabetics vs. 41.22% diabetics). However, it is worth noting that a lower percentage of participants associated with more serious complications, such as amputation at 37.5% (39.52% non-diabetics vs. 60.48% diabetics) and gout at 9.9% (38.64% non-diabetics vs. 61.36% diabetics) with diabetic foot. Figure 2b shows that 53.7% of the participants sought medical care through the primary health care unit (61.1% among the diabetic group vs. 38.9% of the non-diabetic group), followed by the emergency department at 39.8% (56.5% among the diabetic group vs. 43.5% of the non-diabetic group), waiting for next clinic visit at 3.6% (37.5% among non-diabetic vs. 62.5% diabetics), and, finally, using herbal medications at 2.9% (69.2% among non-diabetic vs. 30.8% diabetics).

There were significant differences between those who did not have DM and those who either had DM or their relatives were diabetics in responses to the following questions: “Do you think that diabetes may lead to gangrene in the foot?,” “Do you think that preventing diabetic foot ulcers is more important than treating diabetic foot ulcers?,” and “Do you think it is important to constantly monitor diabetic foot wounds?” (p < 0.05). There were no statistically significant differences between the studied groups in the response to the following questions: “Do you think that diabetes may lead to weakness or loss of sensation in the foot?,” “Do you know that diabetes causes foot ulcers?,” and “Do you think that taking diabetes medications makes the patient vulnerable to diabetes complications?” (Table 2).

**Table 2:** Attitudes of the study participants toward diabetic foot.

Attitude		Total	Do not have diabetes	Have diabetes	Chi-square	P-value
Do you think that diabetes may lead to weakness or loss of sensation in the foot?	I don’t know	112	55 (49.1%)	57 (50.9%)	3.49	0.061
	Yes	333	130 (39.0%)	203 (61.0%)		
Do you know that diabetes causes foot ulcers?	I don’t know	103	48 (46.6%)	55 (53.4%)	1.395	2.37
	Yes	342	137 (40.1%)	205 (59.9%)		
Do you think that diabetes may lead to gangrene in the foot?	I don’t know	70	38 (54.3%)	32 (45.7%)	75.53	0.019
	Yes	375	147 (39.2%)	228 (60.8%)		
Do you think that preventing diabetic foot ulcers is more important than treating diabetic foot ulcers?	I don’t know	128	68 (53.1%)	60 (46.9%)	9.87	0.002
	Yes	317	117 (36.9%)	200 (63.1%)		
Do you think it is important to constantly monitor diabetic foot wounds?	I don’t know	56	39 (69.6%)	17 (30.4%)	20.78	<0.0001
	Yes	389	146 (37.5%)	243 (62.5%)		
Do you think that taking diabetes medications makes the patient vulnerable to diabetes complications?	I don’t know	379	161 (42.5%)	218 (57.5%)	0.866	0.352
	Yes	66	24 (36.4%)	42 (63.6%)		

About three-fifths of individuals who did not have diabetes (60.7%) responded that they “never” perform daily foot checks, while a larger proportion of individuals with diabetes (72.4%) responded that they “mostly” or “always” perform daily foot checks. The chi-square statistic was 28.28, which was highly significant (p < 0.0001). Individuals without diabetes were more likely to respond that they “never” or “rarely” wash their feet carefully, while a larger proportion of individuals with diabetes tended to wash their feet “mostly” or “always.” The chi-square statistic was 20.50, which was highly significant (p < 0.0001), Among individuals without diabetes, a higher percentage responded that they “never” moisturize their feet, while a larger proportion of individuals with diabetes tended to moisturize their feet “mostly” or “always.” The chi-square statistic was 9.73, with a p-value of 0.021. Individuals without diabetes were more likely to respond that they “never” or “rarely” kept their feet away from hot and cold stimuli. On the other hand, a larger proportion of individuals with diabetes indicated that they “mostly” or “always” kept their feet away from hot and cold. The chi-square statistic was 8.97, with a p-value of 0.032. Individuals without diabetes tended to respond that they “never” or “rarely” cared for their nails, while a larger proportion of individuals with diabetes indicated that they care for their nails “mostly” or “always.” The chi-square statistic was 11.59, with a p-value of 0.008 (Table 3).

**Table 3:** Practice of the respondents to keep their feet healthy.

Attitude		Total	Do not have diabetes	Have diabetes	Chi-square	P-value
Daily foot check	Never	112	68 (60.7%)	44 (39.3)	28.28	<0.0001
	Rare	138	52 (37.7)	86 (62.3)		
	Mostly	127	35 (27.6)	92 (72.4)		
	Always	68	30 (44.1)	34 (55.9)		
Wash feet carefully	Never	23	17 (73.9)	6 (26.1)	20.50	<0.0001
	Rare	41	12 (29.3)	29 (70.7)		
	Mostly	142	45 (31.7)	97 (69.3)		
	Always	239	111 (46.4)	128 (53.6)		
Dry the feet and between the toes after each foot wash	Never	93	46 (49.5)	47 (50.5)	4.34	0.227
	Rare	115	43 (37.4)	72 (62.6)		
	Mostly	123	46 (37.4)	77 (62.6)		
	Always	114	50 (43.9)	64 (56.1)		
Moisturize feet	Never	83	46 (55.4)	37 (44.6)	9.73	0.021
	Rare	124	47 (37.9)	77 (62.1)		
	Mostly	129	45 (34.9)	84 (65.1)		
	Always	109	47 (43.1)	62 (56.9)		
Walk barefoot	Never	103	47 (45.6)	56 (54.4)	2.00	0.57
	Rare	187	72 (38.5)	115 (61.5)		
	Mostly	135	56 (41.5)	79 (58.5)		
	Always	20	10 (50.0)	10 (50.0)		
Choose check	Never	100	46 (46)	54 (54.0)	6.86	0.076
	Rare	138	51 (37.0)	87 (63.0)		
	Mostly	116	57 (49.1)	59 (50.9)		
	Always	91	31 (34.1)	60 (65.9)		
Keep the foot away from hot and cold	Never	80	45 (56.3)	35 (43.8)	8.97	0.032
	Rare	117	45 (38.5)	72 (61.5)		
	Mostly	137	51 (37.2)	86 (62.8)		
	Always	111	44 (39.6)	67 (60.4)		
Care for nails	Never	24	18 (75.0)	6 (25.0)	11.59	0.008
	Rare	37	14 (37.8)	23 (62.2)		
	Mostly	117	45 (38.5)	72 (61.5)		
	Always	267	108 (40.4)	159 (59.6)		

## Discussion

The objective of this study was to evaluate the KAP related to diabetic foot among the population residing in Aseer, Saudi Arabia. Participants were divided into two groups. The first group consisted of individuals without diabetes, and the second group included individuals with diabetes or those who were responsible for caring for immediate family members with diabetes. Both groups reported that their primary source of information about diabetic foot was physicians, followed by the Internet. The respondents also identified the most common chronic diseases associated with diabetes as hypertension, obesity, and eye complications. There was a significant difference between both groups regarding their attitude toward the fact that diabetes can cause gangrene, preventing diabetic foot ulcers is more important than its treatment, and the importance of constantly monitoring diabetic foot wounds. There is a strong significant association between diabetes status and the frequency of daily foot checks, the frequency of carefully washing feet, the frequency of moisturizing feet, the frequency of keeping the feet away from hot and cold stimuli, and the frequency of nail care.

Participants accessed knowledge about diabetes and diabetic foot care through diverse channels. Healthcare professionals, specifically physicians, were identified as the predominant source of information for the education of patients. The Internet also emerged as a prominent source. The diverse range of information channels underscores the significance of considering these sources when developing educational interventions and disseminating accurate information to improve diabetic foot care practices. There is a positive correlation between awareness and knowledge of diabetes and self-care behaviors, particularly in high-risk populations, such as individuals with pre-diabetes [13]. In addition to its impact on preventing diabetes in high-risk populations, increased awareness and knowledge of diabetes can also lead to positive results in individuals who already have diabetes [14]. Subramaniam et al. reported that [15] media articles were identified as the main source of information (82.1%), followed by videos/advertisements for health promotion videos/advertisements (77.9%), online websites (58.5%), books (56.5%), healthcare professionals (55.0%), radio (54.4%), public forums (27.7%), and support groups (15.5%).

We found that the correct knowledge across the studied groups was relatively low. However, individuals without diabetes had a lower level of awareness and understanding of the potential complications associated with diabetic foot compared to those who have personal experience with diabetes or are directly involved in caring for individuals with diabetes. We found that a substantial percentage of participants recognized the following complications: swelling, nail destruction, cracks, and wound infection. However, it should be noted that a lower percentage of participants associated with more serious complications, such as amputation. This suggests that there may be room for improvement in educating individuals about the possible severity and consequences of poorly treated or untreated diabetic foot ulcers. In the same vein, Alsaigh et al. [16] found that not only healthcare workers but also the general population have a different level of good knowledge about diabetic food and its care. Interestingly, two-fourths of the respondents consider that the management of diabetic foot is time-consuming, and they may avoid providing medical care for such cases. Many studies reported similar findings, Jia et al. [17] surveyed 1,080 subjects in China, and the findings revealed that 51.6% achieved moderate scores in terms of knowledge about diabetes. Furthermore, 63.9% showed a positive attitude toward diabetes, indicating a favorable mindset. However, concerning practical implementation, 71.4% of the participants obtained poor scores, suggesting suboptimal adherence to foot care practices. In a cross-sectional survey conducted in two Family Health Units in Picos, PI, Brazil, involving 85 individuals of both sexes diagnosed with diabetes, a semi-structured questionnaire on KAP was administered. Regarding foot care, it was observed that 49.4% of the participants lacked knowledge about proper hygiene practices and what to observe in their feet. Similarly, 56.5% of the respondents did not know the correct method for cutting their nails, indicating a lack of knowledge in this area. Regarding attitudes, the study revealed that 80% of the participants expressed a willingness to participate in self-care activities related to foot care [11]. The lack of knowledge and inadequate practice observed may be attributed to the absence of available training and educational programs specifically focusing on diabetic foot care for patients, caregivers, and even the general population [18]. The strategy of community engagement has emerged as a highly effective and impactful approach for advancing knowledge, fostering positive attitudes, and encouraging beneficial practices among patients living with diabetes. By actively involving patients in their personalized treatment plans, there is a notable and significant improvement in their adherence to medical recommendations and overall satisfaction with their healthcare experience [19]. This collaborative and patient-centered approach not only empowers individuals to take ownership of their health but also improves the overall effectiveness of diabetes management, ultimately leading to improved health outcomes and a better quality of life for those affected by this condition [20].

Study strengths and limitations

We should recognize a number of limitations in our study. First, our sample was not chosen using a probabilistic method, which may restrict the generalizability of our findings to the broader population. Second, data collection was performed online through the distribution of a questionnaire on social media platforms. Although this approach allowed for convenient data collection, it introduces the possibility of sampling bias, as not all individuals have equal access to the Internet or engage with social media. However, recent data from the Digital Report on Internet usage in Saudi Arabia suggest that approximately 100% of the population uses social media [21]. Additionally, it is important to note that the data collected for our study pertained to a specific time period (May 2023), and the perspectives of the population may have changed over time. Despite these limitations, our study has several strengths. First, it is the first study conducted in the southern region of Saudi Arabia to assess the KAP of the population regarding diabetic foot ulcers. Second, we included individuals from both healthy and diseased populations, providing a more comprehensive understanding of the perspectives within the population.

## Conclusions

The study findings highlight the need for targeted educational interventions and awareness campaigns to bridge the knowledge gaps and promote consistent and appropriate foot care practices. Additionally, individuals with diabetes or those caring for diabetic individuals should receive continuous education and support to reinforce the importance of preventing diabetic foot ulcers, monitoring foot wounds, and recognizing the potential risks associated with diabetes, such as the development of gangrene in the foot.
